# Are healthy lifestyle behaviours positively associated with the academic achievement of the university students?

**DOI:** 10.30476/JAMP.2019.84152.1129

**Published:** 2020-10

**Authors:** FATIMA YAQUB, CIARA NUTTALL

**Affiliations:** 1 Department of Medicine, Imperial College London, London, UK

Dear Editor,

As 5^th^ year medical students, we took great interest in a recent article concerning healthy lifestyle behaviours and their impact on academic success. Bakouei, *et al*. undertook commendable analysis, subsequently uncovering positive associations, and we thank the authors for their insights ( 1 ). Given our own experiences, we note a few observations and propose further points for consideration.

The first positive association was between the healthy lifestyle behaviour termed ‘spirituality’ and the high achieving academic group. This association is well justified by the authors but is perhaps subject to the culture in which the study was undertaken. The Iranian study sample may differ from other study populations in the spirituality that is represented. Iran has a >99% Muslim population ( 2 ) whilst in Great Britain, for example, the largest religious group is Christianity, followed by ‘no religion’; a group constituting 25-41% of the population in recent years ( 3 , 4 ). The implications this may have on ‘spirituality’ are unknown but a study sample encompassing more varied means of engaging with spirituality may provide alternative results. Further to this, the consequences of being in a majority group versus a minority are well-documented ( 5 ). When spirituality is affirmed by societal norms, stability is achieved, and it is perhaps unsurprising that this creates an environment which enables academic success. Being in a spiritual minority, however, may add undue stress, affecting mental health and thus discouraging academic advancement. 

A second association was found between the higher achieving academic group and those students living at home. Again, this association can be understood by the positive effects of parental monitoring, as described by Bakouei, *et al*. ( 1 ). Whilst this is certainly one explanation, we note that there may be further factors implicated when considering living away from home, namely financial stress. The impact of financial stress in a population accumulating debt has been reported ( 6 , 7 ) and is likely to play more of a role in students living in rental properties. This financial burden may contribute to the association between living situation and academic success. It may prove useful to account for such financial disparity and either study populations of similar economic statuses or incorporate economic status into the analysis.

As highlighted above, the Iranian population findings may differ from elsewhere in the world. The two findings noted above may interact in unidentified ways to produce opposing results elsewhere. For example, in non-religious populations, spirituality may play less of a role but could be substituted by comforts in friendships, in which case living with friends may score more highly in relation to academic success. Whilst plausible, this is merely a hypothesis and indicates that further studies in varying populations are needed to account for the importance of healthy lifestyles in alternative cultures. A healthy lifestyle may vary across the world and amongst individuals. Bakouei, *et al*. do find positive associations in certain domains and as such these domains should be studied further to reduce potential inequalities amongst students with the goal of enabling academic success for all. 
